# Virulence related sequences; insights provided by comparative genomics of *Streptococcus uberis* of differing virulence

**DOI:** 10.1186/s12864-015-1512-6

**Published:** 2015-04-23

**Authors:** Maqsud Hossain, Sharon A Egan, Tracey Coffey, Philip N Ward, Ray Wilson, James A Leigh, Richard D Emes

**Affiliations:** School of Veterinary Medicine and Science, University of Nottingham, Leicestershire, LE12 5RD Sutton Bonington, UK; Sir William Dunn School of Pathology, The University of Oxford, Oxford, UK; DeepSeq, School of Life Sciences, University of Nottingham, Queen’s Medical Centre, Nottingham, UK; Advanced Data Analysis Centre, University of Nottingham, Nottingham, UK

**Keywords:** Mastitis, *Streptococcus uberis*, Comparative genomics, *vru*, *de novo* assembly, CRISPRs

## Abstract

**Background:**

*Streptococcus uberis,* a Gram-positive, catalase-negative member of the family Streptococcaceae is an important environmental pathogen responsible for a significant proportion of subclinical and clinical bovine intramammary infections. Currently, the genome of only a single reference strain (0140J) has been described. Here we present a comparative analysis of complete draft genome sequences of an additional twelve *S. uberis* strains.

**Results:**

Pan and core genome analysis revealed the core genome common to all strains to be 1,550 genes in 1,509 orthologous clusters, complemented by 115-246 accessory genes present in one or more *S. uberis* strains but absent in the reference strain 0140J. Most of the previously predicted virulent genes were present in the core genome of all 13 strains but gene gain/loss was observed between the isolates in CDS associated with clustered regularly interspaced short palindromic repeats (CRISPRs), prophage and bacteriocin production. Experimental challenge experiments confirmed strain EF20 as non-virulent; only able to infect in a transient manner that did not result in clinical mastitis. Comparison of the genome sequence of EF20 with the validated virulent strain 0140J identified genes associated with virulence, however these did not relate clearly with clinical/non-clinical status of infection.

**Conclusion:**

The gain/loss of mobile genetic elements such as CRISPRs and prophage are a potential driving force for evolutionary change. This first “whole-genome” comparison of strains isolated from clinical vs non-clinical intramammary infections including the type virulent vs non-virulent strains did not identify simple gene gain/loss rules that readily explain, or be confidently associated with, differences in virulence. This suggests that a more complex dynamic determines infection potential and clinical outcome not simply gene content.

**Electronic supplementary material:**

The online version of this article (doi:10.1186/s12864-015-1512-6) contains supplementary material, which is available to authorized users.

## Background

Implementation of the five point control measures for bovine mastitis including improved milking practice, post-milking teat disinfection, therapeutic and prophylactic antimicrobial administration, and the culling of persistently infected animals has made significant impact on the control of intramammary infections caused by contagious pathogens [1]. However, these measures are less effective in controlling infections from environmental pathogens, which continue to be a major hurdle in the control of mastitis. *Streptococcus uberis,* a Gram-positive, catalase-negative member of the family Streptococcaceae is an important environmental pathogen implicated in bovine mastitis, accounting for a significant proportion of subclinical and clinical intramammary infections [2]. Mastitis is defined as clinical when abnormality of the udder or secretion is observed, whereas, in subclinical mastitis the udder and the milk appears normal. The economic impact of both subclinical and clinical mastitis in the UK dairy industry is in excess of £200 million/annum with worldwide economic loss estimated at US$35 billion [3]. Control of *S. uberis* through vaccination based strategies therefore has the potential to dramatically improve both the economics of milk production and animal welfare [4]. Development of a vaccine against *S. uberis* has been hampered by a lack of information on the interaction between pathogen and the host [5]. This lack of knowledge is exemplified in the paucity of information on *S. uberis* strains at the genomic level. Whilst over 900 strains of *S. uberis* have been typed using multi locus sequence typing (MLST; http://pubmlst.org/suberis/), only a single genome sequence has been reported, from *S. uberis,* strain 0140J (accession number AM946015), selected as a typical virulent UK strain [6]. The genome of 0140J (1,852,352 bp) is one of the smallest sequenced Streptococcus genomes which range from 1.8 Mb-2.3 Mb [6]. This suggests that through genome reduction, the 0140J genome has become condensed possibly reflecting restricted host-range. It is also possible that the 1,825 protein coding genes of 0140J harbour potential virulence genes which are absent in non-virulent strains, or that loss of accessory genes present in other strains may be associated with the virulence of 0140J.

**Figure 1:**
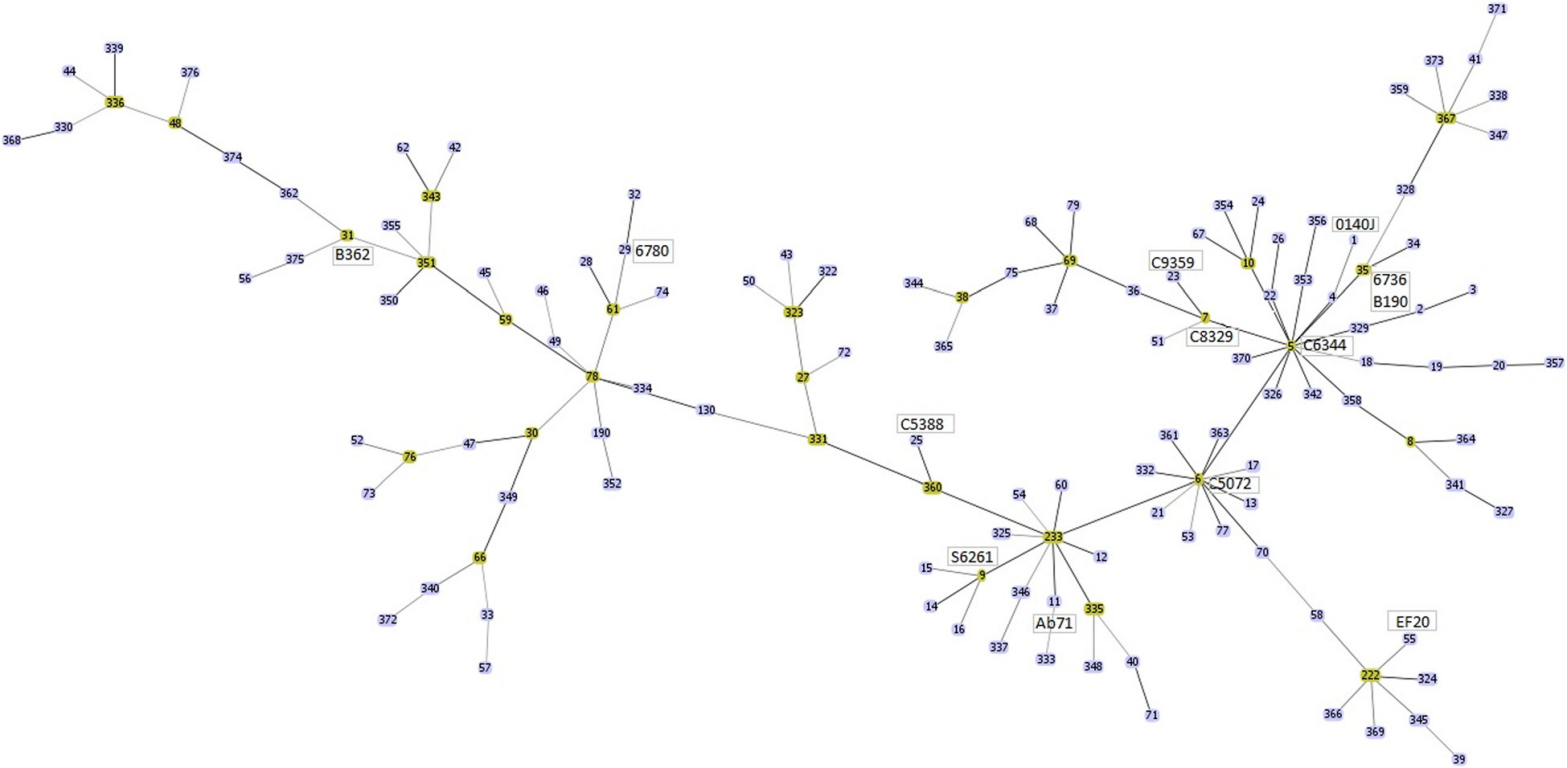
Multilocus sequence typing (MLST) of isolates used in the study. MLST distribution of 13 isolates compared to the other UK strains available in the PubMLST database. MLST profiles of UK *Streptococcus uberis* isolates were downloaded from PubMLST database (http://pubmlst.org/suberis/) and analysed using the goeBURST option of PHYLOViZ [20].

As an initial attempt to rectify this lack of genomic information and to identify the extent of genome variation between *S. uberis* strains, the genomes of an additional twelve strains were determined using high throughput sequencing approaches. The strains selected for sequencing are representative of the currently typed UK strains (Figure 1). Comparison of the predicted gene content was performed to identify the core genome shared by strains and the variable accessory genome between strains. Whilst the simplistic view that presence/absence of single genes or clusters of genes could be used to predict virulence or clinical status is tempting, our analysis suggest that this is not the case. In addition to bacterial factors, complexities such as bacterial population structure, host genetics and host immune status are likely to play a role in the linking of clinical status and bacterial virulence.

## Methods

### DNA isolation and genome sequencing

Bacteria from a range of clinical and sub-clinical isolates (see Additional file 1) were inoculated into Todd-Hewitt Broth and grown at 37°C overnight with DNA extracted from cultures as previously described [7].

### Genome assembly and annotation

Library preparation and sequencing of each strain was conducted at DeepSeq, Queens Medical Centre, Nottingham, UK. 1 μg of high molecular weight *S. uberis* genomic DNA were used to prepare Illumina libraries using the TrueSeq DNA LT Sample Prep Kit (Cat. no, FC-121-2001) as described in TrueSeq DNA sample preparation guide with the following modifications. DNA fragmentation was carried out in covaris S2 using the following parameters: Duty cycle - 10%, Intensity - 5, cycles per burst – 200, Time – 45 seconds, Mode – Frequency sweeping and temperature – 6°C. Gel method was used to size-select adapter ligated DNA to 600-700 bp to generate libraries with insert length of 500-600 bp for the increased MiSeq read length. Sequencing was performed on the MiSeq platform with V2 chemistry (Cat. no, MS-102-2003) to generate 2 × 250 bp paired end reads. The average number of reads per strain was 902,651. Reads were used to generate assemblies using Velvet (version 1.2.10) [8]. Maximum N50 was used as the measure to determine optimal K-mer length using Velvetoptimiser (https://github.com/Victorian-Bioinformatics-Consortium/VelvetOptimiser) a minimum coverage of 10x was selected and the –exp_cov option ‘auto’ was used. CONTIGuator [9] was used to map resulting contigs to the reference genome 0140J for comparative analysis of genomic regions.

Assembled contigs were annotated using the Rapid Annotations using Subsystem Technology (RAST) server [10]. The pan-genome analysis pipeline (PGAP version 1.02) [11] was used for identification of orthologous genes between the twelve sequenced genomes and the reference genome 0140J using the Gene Family (GF) method (50% coverage and with an e-value cut-off 1e-10). Similarity of the samples based in gene presence/absence was conducted using hierarchical clustering. The pvclust package (http://cran.r-project.org/web/packages/pvclust/) was using a correlation distance measure and average agglomeration method.

PILER-CR [12] and CRISPRs web server [13-15] was used for rapid identification and classification of clustered regularly interspaced short palindromic Repeats (CRISPRs). The phage search tool (PHAST) [16] was used to identify, annotate and graphically display prophage sequences within the draft genomes. MUSCLE [17] was used for multiple alignments. The webserver snpTree [18] was used to identify SNP positions trees from the concatenated 1,377 core genes of 13 isolates. PhyML [19] was used for the generation of phylogenetic trees using a GTR model estimated gamma distribution and 4 substitution rate categories. 200 bootstrap replicates were conducted.

Multilocus Sequence Typing (MLST) profiles of UK *Streptococcus uberis* isolates were downloaded from PubMLST database (http://pubmlst.org/suberis/) and analysed using the goeBURST option of PHYLOViZ [20]. Assembled contigs are available at GenBank under the accession numbers provided in Table 1. RAST annotation are available as Additional file 2.

**Table 1:** ***De novo*** **assembly statistics of 12** ***Streptococcus uberis*** **isolates**

**Isolate**	**GenBank accession No.**	**Sequence size**	**Number of contigs (>200 bp)**	**Shortest contig size**	**Longest contig size**	**n50**
EF20	JANW00000000	1933244	18	369	1013731	1013547
6736	JATB00000000	1893525	28	392	424329	386092
6780	JATD00000000	1960858	26	409	404720	340682
Ab71	JATK00000000	1849250	12	449	792968	425420
B190	JATE00000000	1881868	20	450	1043356	1043142
B362	JATC00000000	1912672	31	429	995597	995383
C5072	JATI00000000	1906212	19	431	1021112	1020928
C5388	JATF00000000	1837997	13	409	1030559	1030365
C6344	JATA00000000	1907431	15	431	999672	999478
C8329	JATG00000000	1923969	20	389	830811	455116
C9359	JATJ00000000	1837707	11	450	1030658	1030464
S6261	JATH00000000	1868255	26	429	613122	392636

To determine the stability of pan/core genomes, the pan genome (total number of genes identified within a group of samples) and core genome size (those genes shared by a group of samples) was determined when between 2 and 13 genomes were combined in random order. For each combination size (2…13 genomes) 1000 permutations were conducted.

### Challenge of lactating dairy cattle with *S. uberis* 0140J or EF20

To ascertain the virulence of two *S. uberis* strains, 5 dairy cows aged between 24-30 months were selected for experimental challenge at 4-8 weeks post calving, using a well-established intramammary infection model. Criteria for selection were: absence of signs of mastitis, no history of mastitis during the current lactation and absence of bacteria in milk samples taken 24-48 h prior to challenge with the associated somatic cell count (SCC) below 100,000 cells/ml. Animals were challenged in two mammary quarters after morning milking by infusion of 1 ml of pyrogen-free saline (Sigma) containing approximately 1 × 10^3^ CFU of *S. uberis* 0140J or EF20 prepared as previously described [7].

Following challenge, animals were milked and inspected twice daily. Milk and udder quarters were assessed to determine the severity of disease using predetermined criteria for clinical end points (clotted and discoloured milk and/or udder quarter swollen or causing discomfort on palpation) as previously described [7]. Milk collected from challenged quarters at each milking (up to 48 h) post-challenge was assessed for bacterial numbers and somatic cell counts. The number of viable bacteria present was estimated by plating of each milk sample onto ABA and the number of somatic cells present in milk samples was determined using a DeLaval portable cell counter in line with the manufacturer’s instructions.

## Results and discussion

### General features of *Streptococcus uberis* genomes

**Figure 2:**
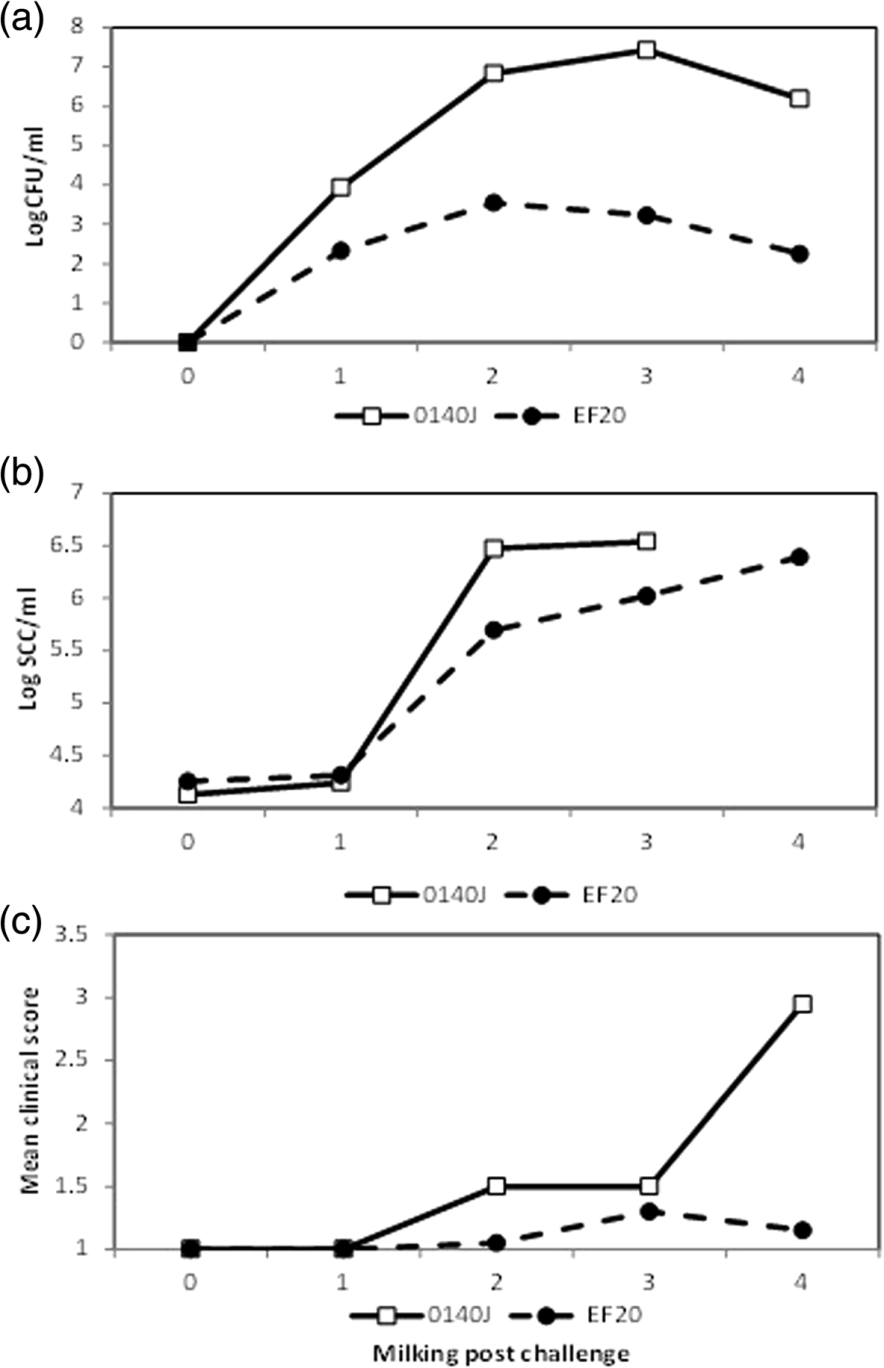
Bacterial isolation, somatic cell count and clinical response following challenge with *S. uberis* 0140J and EF20 in dairy cattle. Geometric mean value obtained after challenge of animals with either *S. uberis* strain 0140J (n = 10) or the EF20 (n = 10). **(a)** Bacterial recovery of *S. uberis* at each milking following challenge, measured by cfu/ml of milk **(b)** Cellular influx at each milking following *S. uberis* challenge, measured by somatic cell count/ml of milk **(c)** Combined clinical scores from clinical manifestations following *S. uberis* challenge. Data is represented as the arithmetic mean of clinical scores given for the appearance of the quarter and appearance of the milk as previously described [7].

Of the 12 strains sequenced, seven were classed as clinical and five sub-clinical isolates based on the status of dairy cow health during strain collection (Additional file 1). The combined length of the assembled contigs range from 1,837,707 bp to 1,960,858 bp (Table 1). This range spans the 1,852,352 bp of the 0140J reference strain and falls within the 1.8 Mb-2.3 Mb predicted by Ward et al [6]. The median size of the genomes from each group differ (clinical median length 1,887,233.5 bp, subclinical median length 1,903,098.5 bp). However, the variation within both groups is large (interquartile range clinical: 74,423.5 bp, subclinical: 55,387.5 bp) and as a result there is no significant difference of total genome size (as measured by total assembled contig size) between the two groups. Therefore this does not support the assuption that clinical strains exhibit a reduced size genome like 0140J. This may reflect the relatively loose definition of clinical vs sub-clinical strains. To investigate the relationship of genome type to virulence phenotype, the representative avirulent strain EF20 was compared in an experimental infection model, to the clinical virulent 0140J strain. All challenged quarters became infected and shed bacteria at detectable levels from the first milking (12 hours post challenge). Those challenged with strain 0140J shed bacteria at 10^4^ cfu/ml of milk 12 h post-challenge) rising to 10^6^-10^7^ cfu/ml of milk by 48 h post challenge (Figure 2a). In contrast, those challenged with strain EF20 shed considerably fewer bacteria ≤ 10^3^ cfu/ml and typically declining to around 10^2^ cfu/ml of milk by 48 h post challenge. The speed of cellular infiltration into the mammary gland in response to infection with either strain was similar, however the magnitude of the infiltration was 10 fold less following challenged with strain EF20. The somatic cell count detected following challenge with strain 0140J was similar to those reported previously for this strain [7,21,22] (Figure 2b). The levels of cellular infiltration and bacterial colonisation for each challenged quarter showed a significant positive correlation (R^2^ = 0.404, P <0.001) over the time-course of the experiment. Acute clinical signs of mastitis (change in milk composition, swollen and inflamed udder quarter) occurred in all animals challenged with strain 0140J (Figure 2c). In contrast, those challenged with strain EF20 showed few, if any, changes in milk composition and/or quarter appearance, thus substantiating and adding detail to the previous data [23] regarding the nature of virulence of these two strains and confirming their suitability for direct comparison at the genomic level in the elucidation of virulence related features.

**Figure 3:**
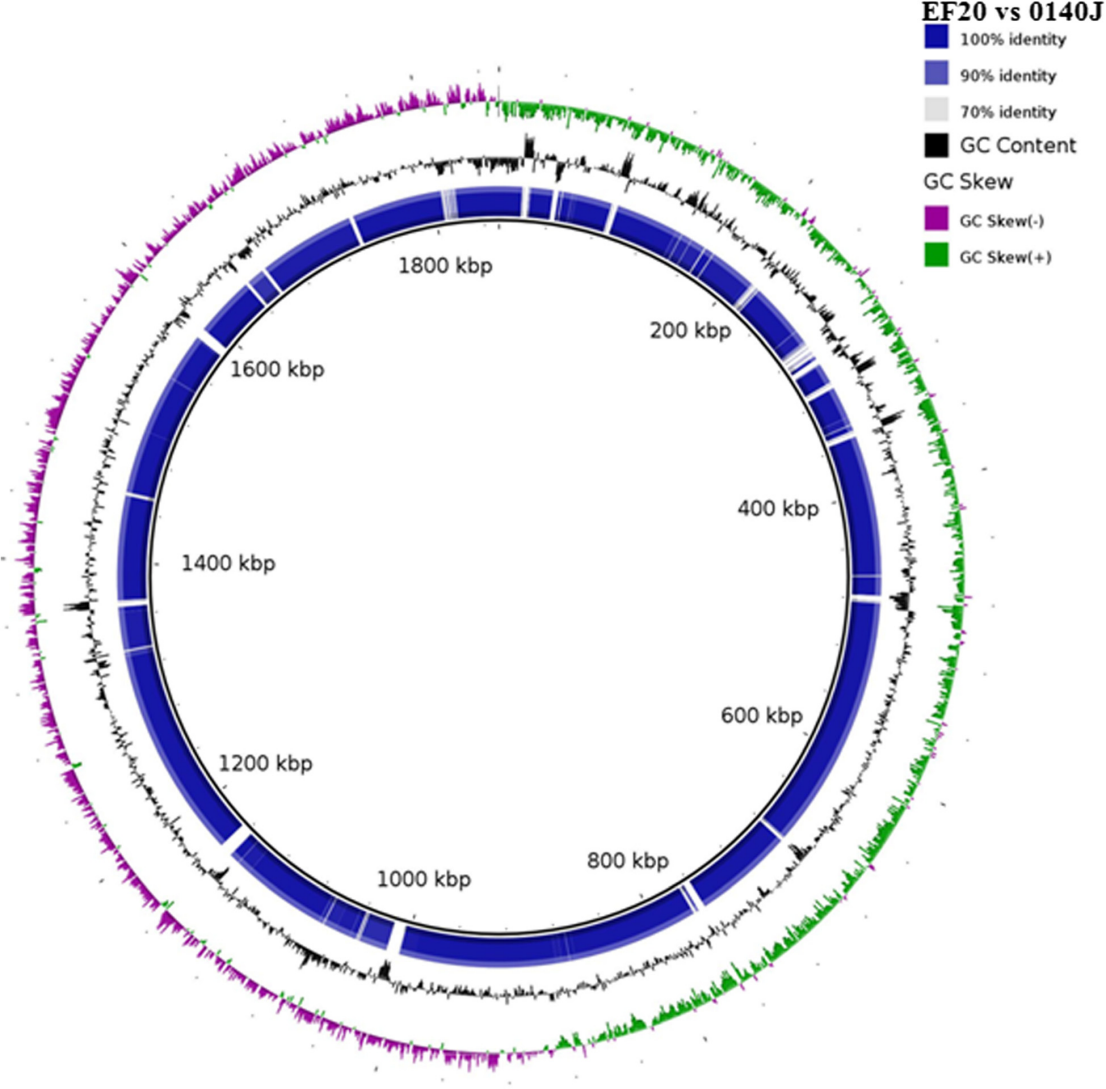
Sequence similarity comparison of 0140J and EF20. The innermost dark circle represents the reference genome 0140J (genome size 1852,352 bp) and the blue circle surrounding the reference genome represents the genome of EF20. Image generated using BRIG [40].

The draft genome of EF20 consists of a slightly larger genome compared with 0140J. The 1,933,244 bp assembled draft genome has a G + C content of 36.5% comparable to the 36.6% G + C content in 0140J. In line with its larger genome, EF20 has an increased number of predicted coding sequences of 1,957 compared to the 1,825 of 0140J. At the gross level, comparative analysis of EF20 and 0140J revealed high-level synteny disrupted by a large number of gene gain/loss and recombination events (Figure 3). The larger predicted gene count in the EF20 genome is not simply addition within EF20 and/or loss in 0140J. Whilst 1,629 annotated genes are common between the two strains, strain specific genes are present in both 0140J (145 genes present in 0140J not in EF20) and EF20 (222 genes present in EF20 not in 0140J) (Additional file 3:orthologous clustering). The copper metabolism operon *SUB1180-SUB1184* was absent in EF20, however, growth comparison studies in restrictive levels of copper does not appear to differentially affect the rate of growth of either strain (results not shown). This may be due to a compensatory gene duplication event of the copper operon located in the region SUB*1462-SUB1464*. Following sequencing of the 0140J strain, a list of suggested virulence genes were proposed for *S. uberis* [6]. Many of these are found in the EF20 genome and appeared to be intact and hence presumably functional, including *SUB1111* (Fibronectin- binding protein), *SUB1273* (Hemolysin like protein), *SUB1154* (C5a peptidase precursor), *SUB0881* (Sortase A), *SUB0145* (Lactoferrin binding protein), *SUB1095* (Collagen like surface-anchored protein), *SUB1635* (SUAM protein), *SUB1785* (PauA Streptokinase precursor) suggesting that the simple presence of these genes is insufficient to explain virulence in *S. uberis*. Comparison of the inferred differences between metabolic subsystems highlights multiple differences (Additional file 4: Subsystem enrichment). Tests for association (*χ*^2^ with Benjamini-Hochberg multiple hypothesis correction p ≤ 0.05) identifies ten subsystems enriched in either 0140J or EF20. Bacterial checkpoint-control-related cluster, F0F1-type ATP synthase, Fructose and Mannose Inducible PTS, Restriction-Modification System, Formaldehyde assimilation: Ribulose monophosphate pathway and Lysine Biosynthesis DAP Pathway subsystems are over-enriched in 0140J compared to EF20. Whereas, D-Tagatose and Galactitol Utilization, Phage replication and Cadmium resistance subsystems are enriched in EF20 compared to 0140J.

**Figure 4:**
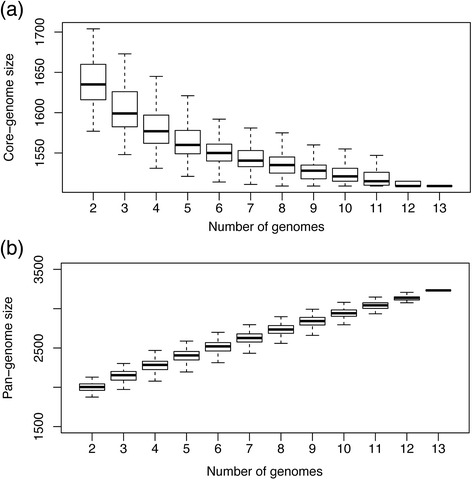
Relationship between gene count and genome size. To determine the stability of pan/core genomes, **(a)** the core genome size (number of common genes within samples) and **(b)** pan genome size (total number of different genes identified within samples) was determined when between 2 and 13 genomes were combined in random order. For each combination size (2…13 genomes) 1000 permutations were conducted. The boxplots represent the median and interquartile ranges of 1000 permutations, whiskers extend to the value extremes.

To further elucidate the complexity of strain differences in *S. uberis*, the genome content of an additional 12 sequenced strains were compared. The sequencing of multiple strains of a given bacteria allows the definition of the core genome, the total number of genes shared by all strains sequenced, this was found to be 1,550 genes between the 12 assembled strains and 0140J (Additional file 3:core_genome) identified in 1,509 orthologous clusters (Additional file 3: RAST_orthologous_clusters). By repeatedly comparing the shared core genome clusters obtained from 1000 random combinations of strains we can see that with two genomes the median shared core genome size is 1,635 genes with a variance (inter-quartile range) of 44 genes. By comparison of 5 assembled genomes the median is 1,560 with inter-quartile range of 29 genes. If 10 genomes are compared the core genome size plateaus at 1,521 genes and the interquartile range is 16 genes (Figure 4a). The identified *S. uberis* core genome contained gene clusters or operons of genes essential for cellular growth and replication including those involved in cell wall and capsule synthesis, cell division, cell cycle regulation and cell signalling, membrane transport (protein secretion systems), RNA/DNA metabolism, metabolism of cofactors, aromatic compounds, amino acids and derivatives (arginine, lysine, threonine metabolism), phosphorus, fatty acid and lipids and carbohydrate uptake and utilization.

### Analysis of the pan genome

The pan genome size (total number of genes within a group of genomes) was determined to give a measure of the relative complexity within the *S. uberis* genomes. With 10-12 *S. uberis* genomes the number of novel genes identified with the addition of an additional genome slows but does not plateau (Figure 4b), suggesting an open pangenome [24]. Together these comparisons of the core and pan genomes suggest that sequencing the relatively small number of strains has captured the majority, but not all of variation of *S. uberis* genomes.

**Figure 5:**
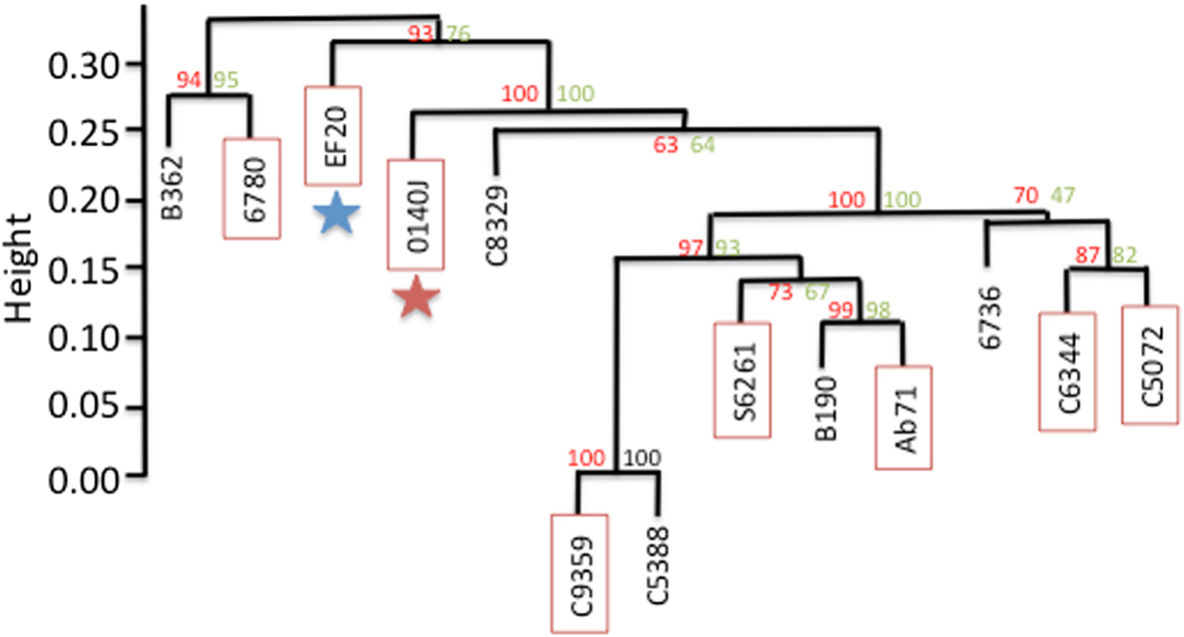
Pan genome clustering of *S uberis* isolates. Similarity of the samples based in gene presence/absence was conducted using hierarchical clustering (pvclust) (http://cran.r-project.org/web/packages/pvclust/). The red star denotes the virulent strain 0140J, blue star, non-virulent strain EF20. Strains initially recorded as clinical isolates are boxed in red.

**Figure 6:**
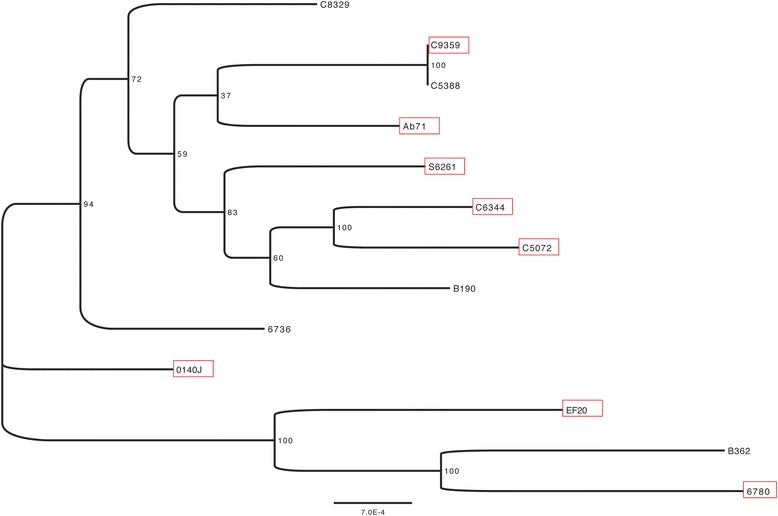
Whole genome phylogeny of *Streptococcus uberis* isolates. A concatenated alignment of 1,377 core genes of 13 isolates was generated by snpTree [18]. The Phi-test [25] was conducted on non-overlapping 500 base windows of this alignment. Following a Bonferroni correction, those windows with significant evidence (*p* < 0.05) of recombination were masked from the alignment and the phylogeny determined by PhyML [19] using a GTR model estimated gamma distribution and 4 substitution rate categories. 200 bootstrap replicates were conducted. Strains initially recorded as clinical isolates are boxed in red.

Direct comparison of all newly sequenced genomes with the available 0140J strain identifies between 115-246 accessory genes present in one or more *S. uberis* strains but not present in 0140J. Following the assumption that the genomes of clinical strains are smaller, the clinical isolates were found to have slightly fewer accessory genes (median 180.5) than the sub-clinical isolates (median 193.5), however again these differences were not statistically significant. In addition, hierarchical clustering of the strains based on their shared accessory genome content, does not group strains by clinical status (Figure 5). Alignment of 1,377 concatenated core genes comprising 1,294,803 nucleotides identified 12,982 variable sites (SNPs). To account for possible recombination affecting the phylogeny the Phi-test [25] was conducted on 500 base windows of the core genome. Following a Bonferroni correction, those windows with significant evidence (*p* < 0.05) of recombination were masked from the alignment (1386 windows total 6,930,000 bases masked) and the phylogeny determined using PhyML as described in the methods. Whilst the bootstrap support was affected, the topology of the tree was unaffected by the masking of possible recombination regions. In agreement with the shared gene content, clustering based on the presence or absence of shared SNPs discriminates the genomes based on ST clonal complex rather than by clinical status (Figure 6).

### Comparison of virulence factors

As with the previous comparison of EF20 and 0140J the reported virulence genes *SUB1111*, *SUB1273*, *SUB1154*, *SUB0881*, *SUB0145*, *SUB1095*, *SUB1635* and *SUB1785* were found in the core genome of all other sequenced strains. Whilst the presence of these genes is conserved in clinical and non-clinical strains, they do exhibit a range of sequence conservation between strains. PauA (*SUB1785*) and Hemolysin-like protein (*SUB1273*) are very well conserved between the 12 strains with only 3 and 5 variable sites respectively in genes of over 800 bp (Table 2a). In contrast, Collagen like surface-anchored protein (*SUB1095*) and Lactoferrin binding protein (*SUB0145*) are highly variable with over 0.4 SNPs/bp. The reference strain 0140J contains a single copy of gene (*SUB*0881) homologous to sortase A (srtA). Mutation of the srtA a transamidase which covalently anchors a subset of proteins to peptidoglycan on the surface of S. uberis has been shown to reduce S. uberis infective potential [7]. This suggests that the sortase-anchored proteins contain one or more virulence factors important for establishment of infection. The sortase-anchored proteins of S. uberis are known to contain a conserved amino acid LPxxG or LPxxxD motif [7]. Using the presence of either of these motifs together with a predicted secretory leader motif, 10 genes (*SUB0135, SUB0145, SUB0207, SUB0241, SUB0826, SUB0888, SUB1095, SUB1154, SUB1370 and SUB1730*) were identified as predicted potential sortase anchored proteins. Nine of these have been confirmed by comparative proteomic analysis [7], whilst *SUB*0241 was not. Sortase anchored genes *SUB0145*, *SUB1095* and *SUB1154* have been shown to be important in infection and have been proposed to be virulence candidates [7]. *S. uberis* mutants in which these genes were inactivated were attenuated in their ability to infect cattle [7]. Of these, *SUB0145* and *SUB1095* are highly variable between strains (Table 2b) suggesting that variation between them is maintained by natural selection in turn suggesting that this may be driven by interaction with the host immune system. Conversely, *SUB1154* is more conserved between strains (0.021 SNPs/bp aligned). Using RAST to transfer annotation from the 0140J reference suggested that *SUB*1095 was unique to 0140J. Since this is an important virulence factor we manually checked whether this gene is truly absent from all other strains. However, using the Rapid Annotation Transfer Tool (RATT) a *SUB*1095 ortholog could be detected in all genomes sequenced suggesting that RAST was unable to annotate this gene due to the highly variable nature of *SUB*1095.

**Table 2:** **SNP distribution of (a) known virulence genes (b) sortase anchored proteins**

**Gene**	**Gene function**	**SNP count**	**Gene size (bp)**	**Alignment size (bp)**	**SNP/bp**
**(a)**					
*SUB*1785	PauA (Streptokinase precursor)	3	861	861	0.003
*SUB*1273	Hemolysin like protein	5	828	828	0.006
*SUB*0881	Sortase A (srtA)	8	759	759	0.011
*SUB*1154	C5a peptidase precursor	73	3480	3362	0.022
*SUB*1635	SUAM protein	52	2637	2637	0.020
*SUB*1111	Fibronectin- binding protein	42	1653	1653	0.025
**(b)**					
*SUB*0135	putative fructan beta-fructosidase precursor	36	3810	3810	0.009
*SUB*0145*	lactoferrin binding protein	552	1819	1352	0.408
*SUB*0207	putative surface-anchored protein	28	1500	1500	0.019
*SUB*0241	putative surface-anchored 2′,3′-cyclic-nucleotide 2′-Phosphodiesterase	48	2478	2457	0.020
*SUB*0826	putative surface-anchored subtilase family protein	208	4492	4418	0.047
*SUB*0888	putative surface-anchored protein	20	852	837	0.024
*SUB*1095*	collagen-like surface-anchored protein	375	1452	935	0.260
*SUB*1154	C5A peptidase precursor	73	3480	3435	0.021
*SUB*1370	putative zinc carboxypeptidase	58	3225	3213	0.018
*SUB*1730	putative surface-anchored protein	128	1191	1020	0.125

*putative virulence gene and sortase substrate.

### Analysis of the has operon

The hyaluronic acid capsule of *S. pyogenes* has been found to play a significant role in the pathogenesis of invasive Group A Streptococcus (GAS) bacteria [26], [27]. *S. uberis* strains isolated from cases of bovine mastitis display variable amounts of hyaluronic acid capsule [6] suggesting that the capsule may be associated with infection. However, Field et al 2003 showed that capsule negative mutants can still cause mastitis [21] and the availability of more capsule in clinical isolates than the environmental isolates [28] may be due to the fact that capsule prevents desiccation in the environment and allows it to persist longer, increasing chances of subsequent infection or even gut colonisation. In *S. uberis* 0140J the arrangement of the hyaluronic acid biosynthetic genes comprising the has operon, differs from the typical “*hasABC”* arrangement common to GAS [28]. H*asA* (*SUB1697*) encoding hyaluronan synthase and *hasB* (*SUB1696*) encoding UDP-glucose dehydrogenase are arranged as in other GAS. However the *hasC* homologue (*SUB1691*), encoding UDP-glucose pyrophosphorylase, is encoded in the reverse orientation and separated from hasAB by approximately 3 kb of genome encoding CDSs thought to be unrelated to capsule biosynthesis [6]. It is unlikely that this arrangement affects capsule production, as in GAS capsule is dependent only upon functional *hasA* and *hasB*, but not *hasC* [29]. All the isolates sequenced here except strain B362 have hasABC in a similar arrangement to that found in 0140J. In nine *S. uberis* strains a paralog of *hasB* (*SUB1027*) was identified. The non-capsular, non-virulent isolate EF20 lacks *SUB1027* and this gene is also missing from isolates B362, 6780 and B190.

**Figure 7:**
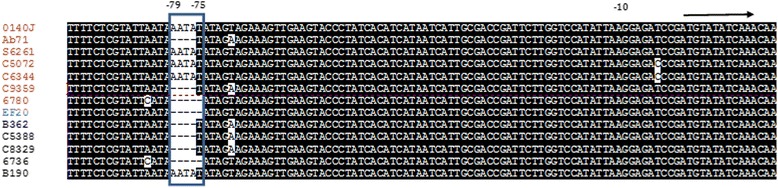
Variation in the *vru* upstream regions. Alignment of upstream region of *vru* gene across 13 strains was generated using Muscle [17]. Boxshade server (http://www.ch.embnet.org/software/BOX_form.html) was used to highlight the high degree of conservation within the aligned region. The position of the initiating methionine codon (ATG) is shown with an arrow. Deletion of TATAA was found in isolates EF20, 6736 and 6780 in position -75 to -79 of *vru* gene. Polymorphism of A to T found in region -79 along with the deletion of four bases TATA in regions -75 to -78 of isolates B362 and C8329. Deletion of four bases ATAA (-76 to -79) was found in 3 isolates Ab71, C5388 and C9359.

*S. uberis SUB0144* is a homologue of the *S. pyogenes* virulence regulatory gene *mga. SUB0144* (*vru*) of *S. uberis* has been found to regulate a number of virulent genes including *hasA* and *hasB1* (*SUB1696* and *SUB1697*), Lbp (*SUB0145*), SclB (*SUB1095*) and PauA (*SUB1785*) and inactivation of *vru* resulted in reduced ability to colonize the mammary gland as well as reduced clinical signs of mastitis compared with the wild-type strain [30]. Moreover, Flores et al have shown that a 12-bp deletion in the VNTR region of *mga* promoter at positions -63 to -75 alters GAS virulence, resulting in asymptomatic carrier phenotype [31]. In *S. uberis* we observe a deletion of five bp in a similar region of vru (position -75 to -79). This deletion was seen in several isolates including the non-virulent strain EF20 and 6736. A four bp deletion was seen in positions -76 to -79 in five isolates Ab71, C9359, B362, C5388 and C8329 (Figure 7). Whilst not perfectly segregating, this deletion was found in most of the sub-clinical isolates and only in three clinical isolates. Hence, variation in this region may play an important role in the regulation of this regulatory gene and in turn influence the host-pathogen interaction.

### Analysis of CRISPR-Cas proteins

**Figure 8:**
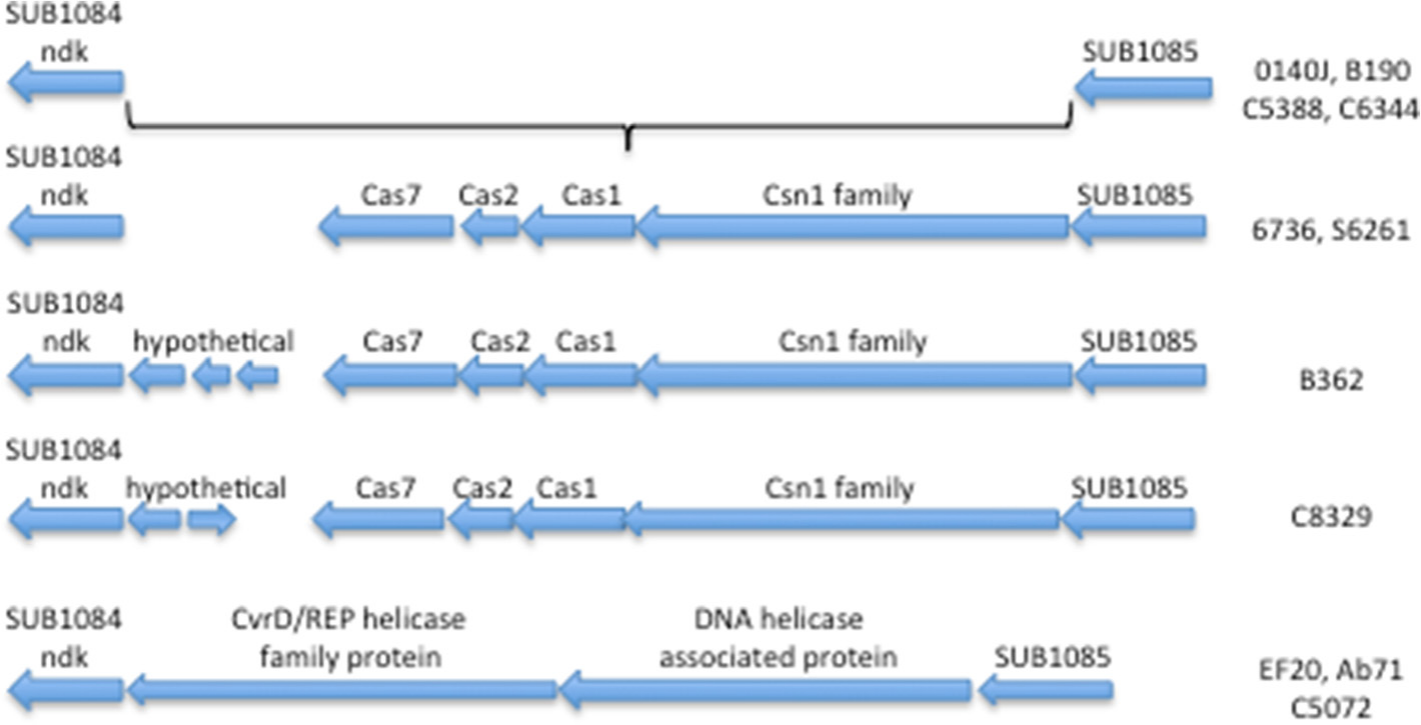
Comparative analyses of Type II CRISPRs regions in different isolates. Relative positions of CRISPR-Cas genes in the 13 isolates. The non-virulent strain EF20 along with the isolates Ab71 and C5072 harbour helicase family proteins instead of CRISPRs regions between genes *SUB1084* and *SUB1085*.

The CRISPR–Cas (clustered regularly inter- spaced short palindromic repeats–CRISPR- associated proteins) identified in approximately 40% bacteria and most archaea, are genomic regions involved in adaptive immunity against invading genetic elements such as viruses [32-37]. CRISPER-Cas genes were detected in all strains except EF20 and 0140J. In strains other than EF20 and 0140J, The type II system [38] which includes the ‘HNH’-type (*Streptococcus*-like) comprising Cas9/Csn1 (a single, large protein) was located in a conserved region between genes *SUB1084-SUB1085* (Figure 8). An additional Type III cas1-cas6 gene set [38] was found in the isolate B362 inserted between genes *SUB*0330-*SUB*0333. In isolates EF20, Ab71 and C5072 an insertion event of two genes, homologous to *Streptococcus pneumoniae* integrative and conjugative elements (ICE) are present in the homologous region between *SUB1084-SUB1085* (Figure 8).

### Analysis of prophage regions

Growing evidence suggests the significant role of prophage regions in the virulence and evolution of many bacteria. For example, lysogeny has been found to contribute to the virulence of a number of organisms including *Vibrio cholerae*, *Salmonella enterica*, *Escherchia coli*, *Clostridium botulinum*, *Corynebacterium diphtheriae*, *Staphylococcus aureus* and *Streptococcus pyogenes* [39]. Analysis of prophage regions shows that among the thirteen isolates seven had intact prophage regions (Table 3). The non-virulent isolate EF20 and subclinical isolate B362 did not have any identified prophage regions. An incomplete prophage region is evident in the region *SUB1818-SUB1840* of the isolate 0140J and this region is variable in most of the isolates. The diversity of these prophage regions may contribute to the adaptation of lysogens to new hosts.

**Table 3:** **Distribution of prophage regions among 13 isolates**

**Strain**	**Region**	**Length**	**Status**	**# CDS**	**Putative phage**	**GC %**	**Location compared to 0140J**
0140J	1		incomplete	28	Lactococcus_phage_bIL311	33.6	*SUB*1818-*SUB*1840
EF20	None						
6736	1	33.5Kb	intact	51	PHAGE_Strept_5093_NC_012753	35.1	*SUB*1470-*SUB*1471
6780	1	35.3Kb	questionable	23	PHAGE_Strept_PH10_NC_012756	40.6	*SUB*1176-*SUB*1187
Ab71	1	12.9Kb	incomplete	15	PHAGE_Lactoc_bIL311_NC_002670	33.1	*SUB*1818-*SUB*1840
B190	1	39.4Kb	intact	56	PHAGE_Strept_pyogenes_315_1_NC_004584	34.9	*SUB*0062-*SUB*0065
B362	None						
C5072	1	47.7Kb	questionable	58	PHAGE_Strept_pyogenes_315_2_NC_004585	39.7	*SUB*1531-*SUB*1532
	2	15.8Kb	incomplete	25	PHAGE_Lactoc_bIL311_NC_002670	32.8	*SUB*1818-*SUB*1840
C5388	1	40.8Kb	intact	57	PHAGE_Strept_P9_NC_009819	37.4	*SUB*1748-*SUB*1452
C6344	1	46.6Kb	questionable	58	PHAGE_Strept_pyogenes_315_2_NC_004585	38.5	*SUB*1531-*SUB*1532
	2	39.6Kb	intact	54	PHAGE_Strept_SMP_NC_008721	36.8	*SUB*1748-*SUB*1452
C8329	1	11.5Kb	incomplete	15	PHAGE_Staphy_phi2958PVL_NC_011344	40.2	*SUB*1183-*SUB*1190
	2	45.7Kb	intact	56	PHAGE_Strept_phi3396_NC_009018	36.5	*SUB*1531-*SUB*1532
	3	37.6Kb	questionable	38	PHAGE_Lactoc_bIL311_NC_002670	35.3	*SUB*1818-*SUB*1840
C9359	1	40.8Kb	intact	57	PHAGE_Strept_P9_NC_009819	37.4	*SUB*1748-*SUB*1452
S6261	1	51.8Kb	intact	68	PHAGE_Strept_TP_J34_NC_020197	36.4	*SUB*1263-*SUB*1270

### Analysis of bacteriocin production

Bacteriocins are proteinaceous antibiotics produced by bacteria, which kill or inhibit the growth of other bacteria, often providing an advantage in competitive colonization environments. Uberolysin is a novel cyclic bacteriocin produced by *S. uberis* encoded by the operon spanning *SUB*0032-*SUB*0036. This operon is absent in EF20 and isolate C9359 but is present in all other sequenced strains. Analysis of the 0140J genome identified five genes encoding putative bacteriocin proteins (*SUB0502, SUB0505, SUB0506, SUB0509 and SUB0512*) [6], of which *SUB502-SUB505* are again absent in EF20 and also isolates B362 and 6780 both of which belong to the ST-86 complex. Whilst bacteriocin production does not define clinical and sub-clinical strains, the absence of almost all bacteriocins in the EF20 genome could put it at a competitive disadvantage with other environmental strains in the dairy cow environment and may reflect (but not explain) it’s non-virulent status.

## Conclusions

The comparison of multiple strains of closely related bacteria provides a valuable resource for the understanding of biological systems. The comparison of 12 newly sequenced strains together with the type 0140J strain of *Streptococcus uberis* allows a first comparison of bacteria isolated from clinical and non-clinical infections and the generation of a draft genome of EF20 strain together with the existing 0140J genome, allows for the first time comparison of two naturally occurring strains of *S. uberis* with defined virulence. The comparison of the strains did not suggest an obvious “smoking gun” gene either present or absent between the virulent or avirulent strains to suggest a previously unknown virulence factor. In addition the genome content did not differentiate between clinical and non-clinical strains. However, it is worth considering that the status as clinical or non-clinical refers to the state of the host animal from which the isolate was obtained, not to the causative agent. For example the proven non-virulent strain EF20 was isolated from a clinical case and hence is named as a clinical strain but this may have been due to other factors such as a co-infection with another bacterial species/strain and importantly, the genetics of the host. Thus, whilst the data here present a detailed comparison of *S. uberis* bacterial strains, to fully understand virulence and causation of disease, we must take a holistic approach encompassing bacteria, host and environment.

### Database submission

Sequence reads and assembled contigs are available at GenBank under accession JANW00000000, JATB00000000, JATD00000000, JATK00000000, JATE00000000, JATC00000000, JATI00000000, JATF00000000, JATA00000000, JATG00000000, JATJ00000000, JATH00000000.

## Additional files

Additional file 1:
**Characteristics of the** ***Streptococcus uberis*** **isolates used in the study.**
Additional file 2:
**Genome annotation of 12 isolates using RAST server.** Downloaded 22/06/14. Additional file 3:
**Identification of orthologous gene clusters between strains.** The pan-genome analysis pipeline (PGAP version 1.02) [11] was used for identification of orthologous genes between the twelve sequenced genomes and the reference genome 0140J using the Gene Family (GF) method (50% coverage and with an e-value cut-off 1e-10). Additional file 4:
**Metabolic subsystem comparison between 0140J and EF20 predicted by RAST server.** Downloaded 22/06/14.
