# Supplementary material for: Virulence related sequences; insights provided by comparative genomics of Streptococcus uberis of differing virulence
Source: BMC Genomics. 2015 Apr 23;16(1):334. doi: 10.1186/s12864-015-1512-6 (PMC4427978; doi:10.1186/s12864-015-1512-6)
Supplement: Additional file 1: — Characteristics of the Streptococcus uberis isolates used in the study. [file 12864_2015_1512_MOESM1_ESM.docx]

**Isolate Year Disease ST Clonal complex**

0140J* 1973 mastitis(clinical) ST1 ST-5 complex

C6344 2002 mastitis (clinical) ST5 ST-5 complex

C5072 2002 mastitis (clinical) ST6 ST-5 complex

S6261 2002 mastitis (clinical) ST9 ST-5 complex

EF20** 1970 mastitis (clinical) ST55

6736 2002 mastitis (sub-clinical) ST35 ST-5 complex

C8329 2002 mastitis (sub-clinical) ST7 ST-5 complex

Ab71 2002 mastitis (clinical) ST11 ST-5 complex

C9359 2002 mastitis (clinical) ST23 ST-5 complex

C5388 2002 mastitis (sub-clinical) ST25 ST-5 complex

B190 2000 mastitis (sub-clinical) ST35 ST-5 complex

6780 2000 mastitis (clinical) ST29 ST-86 complex

B362 2000 mastitis (sub-clinical) ST31 ST-86 complex

**Characteristics of the *Streptococcus uberis* isolates used for whole genome sequencing**. *Clinical virulent reference isolate and **Clinical non-virulent isolate.
